# Human Skin Permeation of Ethoxy- and Propoxypropanol Commonly Found in Water-Based Products

**DOI:** 10.3390/toxics13080675

**Published:** 2025-08-11

**Authors:** Hélène P. De Luca, Jennifer Pache, Philipp Spring, Aurélie Berthet, Nancy B. Hopf

**Affiliations:** 1 Unisanté, University Center for Primary Care and Public Health & University of Lausanne, 1066 Lausanne, Switzerland; helene.de-luca@unisante.ch (H.P.D.L.); jennifer.pache@unisante.ch (J.P.); aurelie.berthet@unisante.ch (A.B.); 2 Department of Dermatology, Lausanne University Hospital (CHUV), 1066 Lausanne, Switzerland; philipp.spring@vidymed.ch

**Keywords:** risk assessment, biomonitoring, skin absorption, organic solvents, exposome

## Abstract

Some propylene glycol ethers (PGEs) have been associated with reproductive toxicity. Ethoxypropanol (PGEE) and propoxypropanol (PGPE) are two common PGEs found in many commercial products. Although skin exposure is frequent when handling such products, no studies have investigated their skin absorption. Neat or aqueous concentrations of PGEs were applied with different concentrations on previously frozen human skin according to OECD guidelines. We also explored the use of frozen skin for skin irritation screening. Our results show that both PGEs readily permeate human skin (permeation coefficients: Kp_PGEE_ = 0.0005–0.002 cm/h; Kp_PGPE_ = 0.0002–0.002 cm/h; rates: J_PGEE_ = 447.5–1075.2 µg/cm^2^/h; J_PGPE_ = 193.9–826.1 µg/cm^2^/h; and time lag: 2–5 h). The permeability rate was four times greater for PGPE diluted in water compared to neat, and double for PGEE. Increasing the water content increased PGEE skin permeation but had no effect on PGPE. Cleaning products contain 1–5% PGEs, and water-based paints 10–50%, thus increasing the potential for skin uptake in consumers. Our skin irritation results were inconsistent, so we conclude that skin irritation cannot be assessed with previously frozen human skin. Future studies should assess the irritation using fresh skin and investigate the risk of health effects from PGEs exposures.

## 1. Introduction

Nowadays, several thousand products contain glycol ethers and are found in paints, cosmetics, cleaning products, inks, and some pesticides. Glycol ethers are divided into two main groups, ethylene glycol ethers (EGEs) and propylene glycol ethers (PGEs). These amphiphilic molecules are used mainly for their solvent properties [1]. Most of these organic solvents are recognized as irritants to both skin and eyes [2], and some are considered reprotoxic and hemotoxic [3]. Indeed, some EGEs were banned from the market because of their reproductive toxicity [4]. They have been replaced by PGEs. Even though PGEs are present in large amounts on the commercial market, very little toxicity data exist. Some are classified as irritants, while others have been reported to produce severe negative neurotoxic effects and induce dizziness and drowsiness (i.e., H336) according to the classification, labeling, and packaging of substances and mixtures (CLP) regulation [5,6]. In fact, central nervous system depression was observed following exposure to high concentrations of PGEs [7,8,9]. Some studies also highlighted the link between chronic exposure to mixtures containing glycol ethers and encephalopathy [10,11].

Consumers and workers are exposed to these substances while handling products containing PGEs. These solvents are easily airborne, so exposures occur through inhalation and skin absorption of the vapor phase as well as direct skin contact. EGEs penetrate the skin barrier readily [12,13,14], but only a few studies have characterized skin permeation following exposures to PGEs despite their possible neurotoxic, reprotoxic, and hemotoxic properties [12,15]. Table 1 summarizes the PGEs skin permeation studies performed in vitro with human skin samples.

Screening chemicals for irritation properties can be performed with human viable skin [15,16]. A more practical approach would be to use previously frozen human skin. The innate immune system is not fully maintained after freezing human skin, but this system is not the only responsible source for triggering irritation. Previously frozen skin can maintain some of the pro-inflammatory properties of the cells and their ability to induce irritation. So far, no studies have assessed whether these properties are sufficiently maintained to screen for chemical irritation.

Two commonly used PGEs in Europe are ethoxypropanol (PGEE, CAS no. 1569-02-4) and propoxypropanol (PGPE, CAS no. 1569-01-3). Both are often present in coating products, while PGEE is present in inks, including finger paints for children [5]. Both are also used in professional and household cleaning products, adhesives, sealants, cosmetics, personal care products, and fuels [6]. Most of these products are water-based formulations containing 10–50% of PGEE and/or 1–5% of PGPE [17]. More than 1000 metric tons of these PGEs are manufactured or imported in Europe each year [5,6]. PGEE and PGPE are sold as isomer mixtures—α (with secondary alcohol group) and β (with primary alcohol group) isomers—and are assumed to follow the same metabolic pathway as other PGEs [18].

The aims of this research are to

1. Characterize the human skin permeation of PGEE and PGPE when applied neat or in aqueous solutions providing the permeation rates (J), lag times (Tlag), and permeability coefficients (Kp) from human in vitro skin permeation experiments using a flow-through diffusion cells system;
2. Characterize the influence of water in the aqueous PGEE and PGPE solutions on skin permeation using a static diffusion cells system;
3. Explore the use of previously frozen human skin to screen these solvents for skin irritation.

## 2. Materials and Methods

The protocol follows the Organization for Economic Co-operation and Development (OECD) Guidelines GD28, TGD428, and GN156 [19,20,21].

### 2.1. Skin

Human abdominal full thickness skin was obtained as surgical waste from the Lausanne University Hospital (CHUV) and the Department of Musculoskeletal Medicine (DAL) Biobank, Switzerland, according to the Swiss ethics in the canton Vaud (CER-VD: Commission cantonal d’éthique de la recherche sur l’être humain, ethical protocol 264/12). The surgeon obtained written consent from the patients before the start of the operation. The collected skin was anonymized, and only sex and age were provided for this study. Human skin was obtained directly after the surgery and immediately dermatomed to a thickness of 800 µm (Acculan^®^II, B. Braun/Aesculap, Sempach, Switzerland) to account for any potential storage effects on its in vitro mass balance [22]. The experiments were run with triplicate skin samples (n = 3) from three different donors giving a total of nine samples for each experiment. The skin samples used for this study were stored at −20 °C for up to four years. The experiments were performed only when a sufficient number of skin samples was available from three different donors.

### 2.2. Flow-Through Diffusion Cell Experiments

The in vitro flow-through diffusion cell experiments were used to assess skin permeation kinetics after neat and aqueous application, separately. The abdominal human frozen skin was thawed for one hour at room temperature and cut into disks (exposed area of 1 cm^2^). These were mounted onto the diffusion cells between the donor and the receptor chamber filled with saline water (0.9% NaCl (aq), 8 mL). Four different experimental conditions were used: application of solutions containing either PGEE or PGPE (neat) and application of aqueous solutions of PGEE or PGPE (50:50 *v*/*v* mixture). Each experiment was performed with triplicate cells from three different donors (n = 12 diffusion cells exposed to PGE per experimental condition) and one control cell per skin donor (n = 3 diffusion cells not exposed to PGE). The trans epidermal water loss (TEWL; VapoMeter wireless, Delfin Technologies Ltd., Kuopio, Finland) was measured for each skin disk to assess skin sample integrity [23]. Skin samples with a TEWL greater than 11 g/m^2^/h were excluded [24]. TEWL was measured again at the end of each experiment to confirm skin integrity throughout the experiment.

The in vitro flow-through diffusion cell experiments were carried out using a rack-mounted system consisting of nine in-line jacketed flow-through diffusion cells (PermeGear^®^ from SES Analytical System, Bechenheim, Germany). A peristaltic pump (Ismatec IPC-N, IDEX Health and Science GmbH, Wertheim-Mondfeld, Germany) operated at a flow rate of 200 µL/min for the first 180 min to ensure that the transition point—when the glycol ethers reached the receptor fluid—was accurately captured for these rapidly diffusing substances. The pump rate was then adjusted to 40 µL/min for the remaining time until 24 h. These collection times and flow rates were set to achieve steady state [15]. The diffusion cell system was equipped with a magnetic stirrer in each cell to ensure a constant and uniform mixing of the substances in the reservoir fluid. The diffusion cells were kept at 32 °C with a heated water bath (Haake SC 100 Digital Immersion Circulator, Thermo Scientific, Newington, NH, USA). A fraction collector (FC 204, Gilson Inc., Middleton, WI, USA) automatically sampled the reservoir fluid at different time points for 24 h, i.e., at 0.5, 1, 1.5, 2, 2.5, 3, 6, 9, 12, 15, 18, 21, and 24 h. PGPE (>95%) and PGEE (>99%) (Chemic Brunschwig AG, Basel, Switzerland) were applied separately onto the skin samples either neat substances or aqueous solutions (Milli-Q Advantage A10 System, Millipore AG, MerckMillipore, Schaffhouse, Switzerland). We applied 500 µL (aqueous application) and 200 µL (neat application) of them to increase the likelihood of reaching steady state permeation conditions [15]. All collected samples were stored at 4 °C after the experiment. Table 2 summarizes the experimental settings for the experiments.

### 2.3. Static Diffusion Cell Experiments

The in vitro static diffusion cell experiments were used to assess the influence of water on PGEE and PGPE skin permeation as well as to evaluate the suitability of frozen skin to test the irritation potentials of these chemicals. The skin irritation experiments had the same set-up as described for flow-through diffusion cell experiments (see Section 2.2), except that these were run in static mode. Each experiment (four different experimental conditions) was performed in triplicate cells from three different donors (n = 12 diffusion cells per experimental condition) and one control cell per skin donor (n = 3 diffusion cells). Again, we applied 500 µL of the aqueous solutions (with 25%, 50% or 75% of solvents) and 200 µL of neat substance. The reservoir fluid (saline water) from each diffusion cell (n = 24) was collected after 24 h post-application and stored at 4 °C after the experiment for chemical analysis (Section 2.4) whereas skin samples were kept for histopathological analysis for irritation assessment (Section 2.6). Table 2 summarizes the experiments.

### 2.4. Chemical Analysis

The concentrations of parent compounds were quantified by gas-chromatography tandem mass spectrometry (GC-MS/MS). The GC (TRACE 1310, Thermo Scientific, Thermo Fisher, Ecublens, Switzerland) was equipped with an autosampler (TriPlus RSH, Thermo Scientific, Thermo Fisher, Ecublens, Switzerland) and a capillary column (ZB-FFAP; 20 m × 0.18 mm i.d., 0.36 µm film thickness, Zebron, Phenomenex, Basel, Switzerland) and operated in headspace mode (HS). The MS/MS (TSQ 8000 Evo, Thermo Scientific, Thermo Fisher, Ecublens, Switzerland) was operated in electron impact ionization mode. The HS glass vials (20 mL) were incubated for 10 min at 90 °C before the solid phase microextraction (SPME fiber carboxen/polydimethylsiloxane, CAR/PDMS, 85 µm, Superlco, Buchs, Switzerland). The extraction time of PGEE was four minutes and five minutes for PGPE. The SPME fiber was desorbed at 250 °C and then transferred into the column in splitless mode. The split flow was 20 mL/min. The GC oven temperature program ran from 40 °C to 125 °C with a rate of 6 °C/min. The retention time for PGEE was 5.94 min and 7.53 min for PGPE. The limits of detections (LODs) for PGEE and PGPE were 0.045 µg/mL and 0.035 µg/mL, respectively, and the limits of quantifications (LOQs) for PGEE and for PGPE were 0.135 µg/mL and 0.105 µg/mL, respectively. Data processing and acquisition were performed using the Thermo Scientific Chromeleon software (2025).

### 2.5. Statistical Analysis

The results from flow-through and static diffusion cell experiments were compared using the RStudio v1.6.0 software [25]. Two-sample *t*-tests or Wilcoxon tests were performed to test the difference between permeability rates obtained with flow-through diffusion cell experiments. Regarding static diffusion cell experiment results, Kruskal–Wallis tests were used to test the difference between the different experimental conditions, i.e., undiluted or diluted in 25%, 50%, or 75% of water.

### 2.6. Skin Irritation Analysis

The skin disks were immediately immerged in formalin for histopathology after end of experiment as well as small skin samples from the original piece. The skin samples were classified into four irritation categories: normal, focal spongiosis/apoptosis, diffuse spongiosis/vacuolization, and epidermolysis. The method used to evaluate skin irritation has been described previously in [16].

### 2.7. Data Treatment

The cumulative amount of PGEE and PGPE in the receptor fluid (µg/cm^2^, y-axis) was plotted separately over time (hours, x-axis), and the permeability rate (J), the lag time (Tlag), and the permeability coefficient (Kp) were determined from the generated permeability curve. J (µg/cm^2^/h) was calculated as the slope of the steepest linear part of the curve (apparent steady state), and Tlag (h) as its x-axis intercept. Kp (cm/h) was calculated by dividing J with the chemical concentration applied to the skin when diluted in water and with the glycol ether’s density when applied neat [26,27]. The maximal internal dose in the bloodstream was estimated using the permeability rate obtained for the experiments and calculated as follows:

IDmean = J [µg/cm^2^/h] × SA [cm^2^] × t [h] × P, (1)

where IDmean is the mean internal dose of PGEE or PGPE after skin exposure, J is the experimental permeability rate, SA represents the surface area exposed to the solvent, t is the time of exposure, and P represents the average percentage of PGEs in commercialized products. We used the J from the aqueous experiments because PGEs are mostly present in water-based products. The surface area exposed was set to 10.4 cm^2^ for the hands alone, and 14.2 cm^2^ when including the forearms [28]. The total blood volume used for the blood concentration calculation was 5 L [29]. In Switzerland, paint products on the market for both professional and consumer uses contain, on average, 22.3% of PGEE and/or 5.9% of PGPE [17].

### 2.8. Mass Balance

A mass balance was performed only for flow-through diffusion cell experiments following the OECD guidelines, where the mean recovery should be 100 ± 10% [20]. The following components of the test system were analyzed to determine recovery: donor chamber, skin, and receptor chamber. The donor chamber was rinsed three times with 500 µL of water (donor chamber samples), the skin disk was sonicated three times for five minutes in water (skin samples), and the remaining fluid receptor chamber was collected (receptor chamber samples). If liquid was present in the donor chamber at the end of the experiment, it was also collected and analyzed with the donor chamber samples. PGE concentrations were quantified in all mass balance samples by GC-MS/MS, i.e., donor chamber rinsing water, water collected after sonication of skin, and remaining fluid receptor chamber. Mean concentrations of PGEE and PGPE were calculated for all cells in both conditions (neat or aqueous), and the results were reported as the percentage of the applied dose.

## 3. Results

### 3.1. Flow-Through Diffusion Cell Experiments

**Figure 1:**
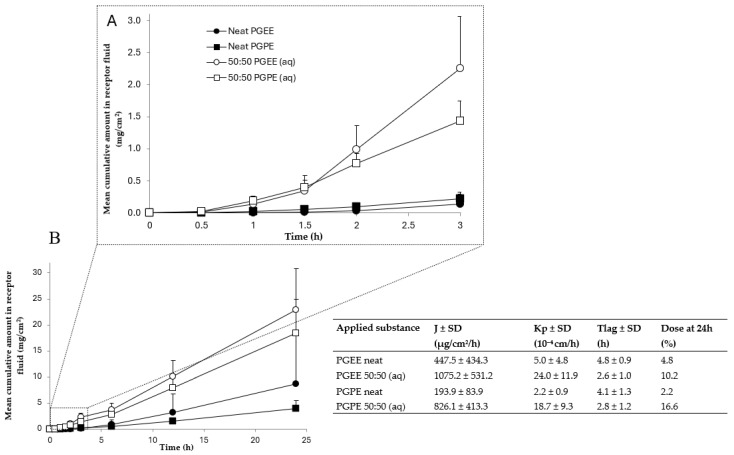
Mean cumulative amount in the receptor fluid (mg/cm^2^) (y-axis) of neat or aqueous PGEE and PGPE over time (x-axis) in a flow-through diffusion system (*n* = 3, from three different human donors) with in vitro skin permeation parameters (J, Kp and Tlag) for PGEE and PGPE estimated from their permeability curves. Skin permeation of studied glycol ethers during the first 3 h of exposure (**A**) or 24 h of exposure (**B**). The last column of the table represents the percentage of the cumulative dose quantified in receptor fluid samples after 24 h.

Figure 1A illustrates the permeability curve for neat and aqueous PGEE and PGPE over the first three hours of exposure, whereas Figure 1B shows the curves over a 24 h period. PGEE and PGPE reached a steady state under all experimental conditions. Human skin permeation for both PGEs was statistically significant when they were applied diluted in water (PGEE *p*-value = 0.014; PGPE *p*-value = 0.026). No significant differences were observed between propylene glycol ethers under the same experimental conditions, i.e., neat and diluted 50:50 application. The skin permeation was fast for both PGEE and PGPE. They were quantified in the receptor fluid as soon as 30 min after application (Figure 1A). In Figure 1, the table summarizes the J, Tlag, and Kp results for PGEE and PGPE according to the different conditions of exposure. The permeability rate, the permeability coefficient, and the percentage of cumulative dose at 24 h were all greater for aqueous PGEE and PGPE, which also showed a shorter Tlag. The Tlag was 2.5–3 h for aqueous PGEs and 4–5 h for neat (Figure 1).

### 3.2. Static Diffusion Cell Experiments

**Figure 2:**
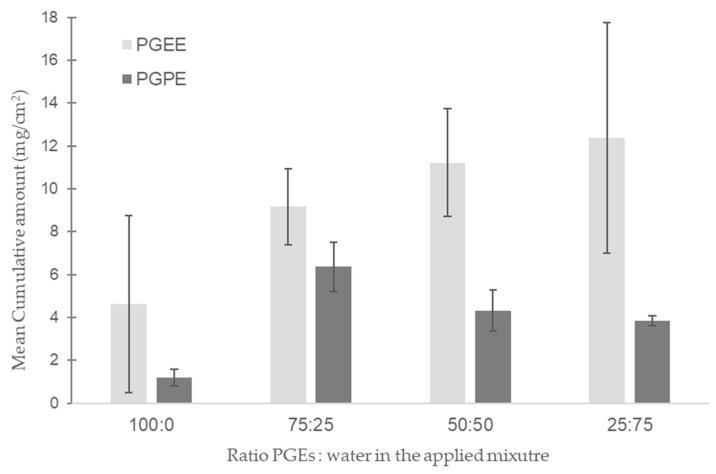
Skin permeation experiment of PGEE and PGPE through static diffusion cell system (*n* = 3). Mean cumulative amount after 24 h of exposure (µg/cm^2^, y-axis) according to different concentration of propylene glycol ethers (x-axis). PGEE and PGPE were undiluted or diluted in 25%, 50%, or 75% of water and applied on previously frozen human skin (*n* = 3 cells for each condition).

Figure 2 represents the cumulative mean concentration of PGPE and PGEE quantified in the receptor fluid after 24 h of exposure for static diffusion cell experiments. The PGEs were applied neat or diluted in water at different percentages (0, 25, 50, or 75% of water). The cumulative mean concentration was statistically significant between experimental conditions only for PGPE (*p*-value = 0.03). The permeation through previously frozen human skin was greater for PGEE compared to PGPE, but it did not reach statistical significance. The mean amount of PGEE increased with the percentage of water in the applied mixture following a logarithmic fit (y = 5.65ln(x) + 4.87; R^2^ = 0.99). The in vitro skin permeation of PGPE did not follow the same trend. Skin permeation did not increase with increasing water concentration; it was low after neat application of PGPE and reached a maximum when diluted with 25 % water (75:25), followed by a decrease at higher water concentrations (Figure 2).

### 3.3. Skin Irritation Analysis

Focal spongiosis and epidermolysis were observed in 20% of the skin samples prior to solvent exposure. No significant effect between controls and exposed samples was observed in skin irritation analysis. Overall, these data were inconsistent with respect to irritation.

### 3.4. Mass Balance

Table 3 summarizes the mass balance results for the flow-through diffusion cell experiments. Total recovery was low (<20%) when neat substance was applied on the skin compared to higher recovery with aqueous application (<50%). No substance remained on the skin at the end of the experiments.

## 4. Discussion

### 4.1. Flow-Through In Vitro Skin Permeation

Our results show that PGEE and PGPE permeated previously frozen human skin within 30 min when aqueous PGEs were applied and after two hours when applied neat (Figure 1). In addition, the high permeation rates indicate that PGEE and PGPE readily pass through human skin. The permeability coefficients we found for PGEE and PGPE correspond to other PGEs reported in the scientific literature, such as propylene glycol methyl ether (PGME) and propylene glycol butyl ether (PGBE) [12,13,14,15,30]. There is little in vitro or in vivo human skin permeation data for the majority of PGEs and none for those included in this study. However, all existing results reported that molecules of this family easily and rapidly permeate the skin barrier with their high J and short Tlag (Table 1). Therefore, skin contact is an important route of human exposure to these molecules, and water appears to enhance their ability to permeate skin. Because products containing PGEs are available on the commercial and professional markets, both consumers and workers can come into direct skin contact with these substances. According to our results, it becomes crucial to have toxicological and exposure data concerning these organic solvents [31], especially given the rising use of PGEs in water-based products [32,33].

### 4.2. Mass Balance

The total recovery rates obtained in flow-through diffusion cell experiments vary between 10% and 45% (Table 3). These values are substantially lower than the standard values outlined by OECD guidelines [20]. No mass balance data exist in previous skin permeation studies of PGEs (Table 1). Our low recovery could potentially be explained by the volatile properties of these PGEs, by metabolite formation, or by their accumulation in the skin. No substance remained on the surface of the skin at the end of our experiments under any of the tested conditions. Given that no data exist for assessing evaporation of these substances from skin, we assessed the percentage evaporated experimentally. The evaporation of these glycol ethers was assessed at room temperature using glass vials with a diameter corresponding to the diameter of diffusion cells and with the same volume as we tested in the skin permeation experiments. These vials were weighed before and after 24 h to assess the loss of mass due to evaporation. We observed no evaporation (the vials had the same weight before and after 24 h) for neat PGEs (<2%) and between 12% and 13% evaporation for aqueous solutions of PGEs. Hence, we recommend that future studies investigate the possible evaporation of glycol ethers. Occlusion studies may be suitable; however, these may also modify skin permeation kinetics when compared with non-occluded skin [34]. Although these evaporation evaluations are only estimates, they support the differences observed in mass balance between neat and aqueous applications. However, they only partly explain the low recovery. An alternative hypothesis is that PGEs are metabolized in skin. The metabolism of the β isomer is characterized by two successive oxidative pathways involving alcohol (ADH) and aldehyde dehydrogenases (ALDH), respectively, and resulting mainly in the formation of carboxylic acid metabolites [7]. The main localization of these enzymes is the liver, but they are also expressed in other tissues such as the skin. ADH and ALDH are present in the epidermis of human skin and are still active after skin freezing [35]. One study investigated the formation of glycol ether metabolites after in vitro human skin permeation [36,37]. After 24 h, they quantified approximately 0.03% of the applied dose that had been metabolized during its passage through the skin. Given the small metabolic conversion, the formation of PGE metabolites in our samples seem possible but can only explain a fraction of the low recovery. We believe that these mass balance results are likely explained by the inefficient extraction of PGEs remaining in the skin. Our extraction efficiency was not evaluated, and extracting such substances is a challenging process. Therefore, we hypothesize that the actual percentage of the substance stored within the skin is probably much greater than what we measured.

### 4.3. Static In Vitro Human Skin Permeation

In vitro skin permeation was greater for PGEE compared to PGPE in our flow-through diffusion cell experiments. This was corroborated by the results of the static diffusion cell experiment. Two main parameters govern skin absorption capacity, namely molecular weight and log octanol-water properties [38]. A high molecular weight reduces the diffusion of a substance through the stratum corneum and the rate of the permeation [39]. Differences in molecular weight accounts for the higher permeability rate of PGEE compared to PGPE [26,27]. LogP is an indicator of the lipophilicity of a substance. Passive diffusion dominates for LogP values below 0.5 [40]. LogP for PGEE and PGPE are 0.2 and 0.7, respectively [26,27]. The higher human skin permeation observed for PGEE over PGPE can thus partly be explained by passive diffusion, which is favored for PGEE according to its LogP value.

Furthermore, according to Fick’s law, we expected skin permeation in the static experiments to increase with increasing percentage of water (vehicle) based on our flow-through experiment results. This hypothesis was true for PGEE but not for PGPE (Figure 2). PGEs are solvents and have dissolving properties on lipids. The upper skin layer (i.e., the stratum corneum) consists of corneocytes surrounded by a lipid-rich extracellular matrix [38]. The stratum corneum is the principal barrier to chemical entry. Thus, PGEs can dissolve lipids present in the first skin layer, decrease the barrier resistance, and allow the passage of chemicals through the skin. PGEs also have a high aqueous solubility at room temperature, and the addition of water may increase the permeability rate [41]; these are outcomes that we observed for the PGEE skin permeation. However, these observations were not true for PGPE. The mean cumulative amount of PGPE quantified in the receptor fluid after 24 h first increased with 25% of water in the solution, then decreased as the water percentage increased further (Figure 2). In 2007, Traynor et al. studied the dermal permeation of water-miscible compounds, including ethylene glycol butyl ether (EGBE). They observed three behaviors depending on the proportion of water. At water concentrations below 25%, the permeability rate increased with EGBE concentrations. The relationship between water thermodynamic activity and solvent concentration was linear. The permeability rate remained constant at intermediate water percentages—greater than at low water percentages—but decreased with increasing EGBE concentrations above 50% water [42,43]. These results are comparable to those observed for PGPE skin permeation in our study. The driving force for PGEE human skin permeation appears to be the solvent concentration according to Fick’s law’s prediction; however, for PGPE permeation, the driving force seems to be the thermodynamic activity of the solutions [42,44].

### 4.4. Effect of Water in Human Skin Permeation

Our results also show that the skin permeation of these organic solvents differs when they are applied diluted in water. We observed that J was increased to half when aqueous PGEE was applied and more than four times quicker for aqueous PGPE compared to neat solution (Figure 1). These results indicate that PGPE and PGEE permeate the skin faster when they are applied diluted in water. Water from perspiration or water-based products, for example, is known to accelerate a solvent’s skin permeation [30,43]. In 2004, Venier et al. studied the impact of water on the skin permeation of dipropylene glycol methyl ethers (DPGME). They aimed to reproduce the actual conditions of use in an industrial environment. The permeability coefficients of neat substances were lower than for aqueous solutions [13]. These results are similar to ours. PGEs have a dehydrating effect on human skin, which can contribute to a low permeability rate. The addition of water in the mixture and the rehydration of the skin can increase the absorption of the compounds [41,43]. An increase in permeability rate can also be due to an alteration in the skin barrier properties of the stratum corneum. The stratum corneum is essentially composed of lipids. The application of PGEs on skin can disrupt the stratum corneum due to their dissolving properties and easily permeate the skin. Another reason for an increased permeability rate can be a higher degree of partitioning into skin layers. In fact, PGEs are amphiphilic molecules, soluble in hydrophilic regions of the different skin layers (i.e., the epidermis and the dermis) [43].

### 4.5. Irritation

Glycol ethers are well known to cause skin irritation [5,6,45,46]; however, our results showed an inconsistent skin irritation pattern in both the control and the solvent-exposed skin samples, even though the TEWL measurements were under 11 g/m^2^/h for all used skin samples, indicating a good integrity of the skin barrier function. We expected irritation effects similar to those reported in published in vitro studies using fresh human skin to assess skin irritation [15,16]. Skin quality varies according to different factors, such as age, colors, skin diseases, and storage conditions, which can have an impact on irritation effects due to the exposure of solvents. However, no studies have investigated the effect of freezing temperatures (−20 °C versus −80 °C) or storage duration in the freezer on skin irritation. Based on our observations, we do not recommend using previously frozen skin; instead, fresh [16] or synthetic human skin [47] should be used for testing the irritation properties of chemicals in vitro. Further investigations are necessary to assess the irritation of propylene glycol ethers.

### 4.6. Workplace Scenario

Recreating a workplace scenario, the mean internal dose after one workday for a worker in contact with products containing PGEE or PGPE was estimated based on our results. Mixing tasks are common in companies that use paint products, such as those in the construction and car repair industries. This task usually involves opening containers and mixing large quantities of paint. Workers’ hands, and occasionally forearms, are often contaminated by paint splashes and contact with various painting equipment [48]. Based on our in vitro results and assuming dermal exposure only, after one 8 h workday, with a SA of 10.4 cm^2^ and PGEs content of either 22.3% or 5.9%, the mean estimated internal dose for a worker performing a paint mixing task without wearing gloves is 8.3 mg of PGEE (see Equation (1)). This corresponds to a blood concentration of 1.7 mg/L. Under the same conditions, if the worker is exposed to products containing PGPE, the mean estimated internal dose after one workday is 0.9 mg, corresponding to a blood concentration of 0.2 mg/L. The addition of water to the mixture, for example, from perspiration or from the water-based product itself, increases the estimated internal dose to 19.9 mg (3.9 mg/L) for PGEE exposure and 4.1 mg (0.8 mg/L) for PGPE exposure. Some paint products may contain both PGEs mixed with other chemical substances; however, mixtures were not considered in this scenario. Further studies are necessary to investigate the effects of chemical mixtures on glycol ether skin permeation. Furthermore, PGEE and PGPE are volatile organic compounds, so workers can be exposed not only through direct skin contact but also via skin vapor absorption through the skin or inhalation. Therefore, the blood concentrations estimated in our workplace scenario would likely be higher under real working conditions involving all routes of absorption. Since no biological reference values are currently available for these compounds, it is not possible to estimate the potential risks associated with exposure at these blood concentrations. However, due to the high skin permeation of these compounds, workers and consumers must wear appropriate protective equipment to limit their exposure.

### 4.7. Limitations

Our study was conducted in accordance with the OECD guidelines, with some deviations. Only three donor skins were used (instead of the recommended four–six donor skins) due to limited availability during the COVID-19 pandemic. However, the low inter-donor variability supported the reliability of the data. We did not investigate the metabolic transformation of PGEs, and while the absence of metabolites may have contributed to the low mass balance, this is unlikely, given the limited metabolic activity of frozen skin and the low proportion of the metabolizable primary alcohol isomer in technical-grade PGEE and PGPE. Extracting amphiphilic molecules such as glycol ethers from human skin is inherently difficult and was not evaluated in this study. One approach to quantify substances retained within the skin is complete tissue digestion [14,43], which typically requires radiolabeled compounds. Since PGEs are not currently available in a radiolabeled form, extraction efficiency likely affected our mass balance results. Additionally, some loss of propylene glycol ethers may have occurred due to adsorption onto the plastic or glass surfaces of the experimental apparatus [49], which could only be confirmed using radiolabeled substances. Although frozen skin may exhibit increased permeability, it is accepted under OECD guidance when properly stored and handled, as was the case here, with barrier integrity confirmed via TEWL. While we did not test the 1–5% concentration range typical of cleaning products, our 25% dilution condition is considered representative. A concentration-dependent trend was observed for PGEE but not for PGPE, suggesting compound-specific behavior. Despite these limitations, the study provides robust and relevant data for risk assessment of PGEs in products, supporting the need for further refined studies.

## 5. Conclusions

PGEs permeate human skin rapidly—within 30 min for aqueous PGEs, while within two hours when applied neat. Both substances also had high permeation rates (J = 0.2 to 1.1 mg/cm^2^/h). The greater the amount of water, the faster PGEE permeated the skin; however, this was not observed for PGPE skin exposure. Our results showed that these PGEs are, like other glycol ethers, able to cross the skin barrier and reach the bloodstream. However, water plays a role as an enhancer in the skin permeation of these substances, although these relationships are not fully understood. Our results showed inconsistent irritation patterns with solvent exposures in previously frozen skin samples. We therefore recommend not using previously frozen skin samples for in vitro skin irritation screening. Given the rapid dermal absorption of PGEs and the unknown health effects associated with these organic solvents, we recommend protecting workers from skin exposure during the use of products containing these substances, as there is a lack of data on their toxicity.

## Figures and Tables

**Table 1:** Summary of previous in vitro skin permeation studies (from most recent to the oldest) of different propylene glycol ethers in humans, including propylene glycol methyl ether (PGME), propylene glycol butyl ether (PGBE), dipropylene glycol methyl ether (DPGME), and propylene glycol methyl ether acetate (PGMEac).

Tested Chemical	Skin Thickness (mm)	Skin State (Frozen/Fresh)	Receptor Fluid (mL)	Cell Size (cm^2^)	Temperature (°C)	Applied Dose (mg/cm^2^)	Duration (h)	Kp ± SD (10^−3^ cm/h)	J ± SD (µg/cm^2^/h)	Tlag ± SD (h)
Berthet et al., 2020 [15]										
PGME	Dermatomed (0.8)	Frozen	NaCl 0.9% (12)	1.77	32	109.6	24	0.18 ± 0.06	174 ± 62.2	0.88 ± 0.05
PGME	Dermatomed (0.8)	Fresh	NaCl 0.9% (12)	1.77	32	109.6	24	0.04 ± 0.04	40.4 ± 37.7	1.18 ± 0.16
PGBE	Dermatomed (0.8)	Frozen	NaCl 0.9% (12)	1.77	32	100.0	24	0.05 ± 0.02	44.1 ± 16.2	0.86 ± 0.23
PGBE	Dermatomed (0.8)	Fresh	NaCl 0.9% (12)	1.77	32	100.0	24	0.009 ± 0.002	7.93 ± 1.57	0.89 ± 0.13
Venier et al., 2004 [13]										
DPGME	Full thickness (1)	Frozen	NaCl 0.9% (14)	3.29	32	57.8	8	0.11 ± 0.04	106.3 ± 37.6	1.51 ± 0.48
Wilkinson and Williams 2002 [14]										
PGME	Dermatomed (0.5)	Frozen	* SHC/G (0.4)	0.64	32	286.3	5	na	4.3 ± 0.5	0.68 ± 0.09
Larese et al., 1999 [12]										
PGME	Full thickness	Frozen	NaCl 0.9% (15)	3.14	32	58.3	4	0.51 ± 0.13	472 ± 120	0.55 ± 0.05
PGMEac	Full thickness	Frozen	NaCl 0.9% (15)	3.14	32	61.7	4	0.06 ± 0.006	59 ± 44	0.5 ± 0.05
PGBE	Full thickness	Frozen	NaCl 0.9% (15)	3.14	32	56.1	4	0.02 ± 0.005	17 ± 5	0.62 ± 0.03

* SHC: Sodium hydrogen carbonate; G: Gentamicin.

**Table 2:** Summary of the experimental conditions for diffusion cells with frozen skin at a thickness of 800 µm for 24 h.

Substance	Diffusion Cell Type	Flow Rate (µL/min)	Applied Substance Concentration (%)	Volume (µL)	Amount (mg)
*Ethoxypropanol (PGEE)*					
Neat	Flow-through	200 (0–3 h); 40 (3–24 h)	100	200	179.2
Neat	Static	-	100	200	179.2
Aqueous	Static	-	75	500	336
Aqueous	Flow-through	200 (0–3 h); 40 (3–24 h)	50	500	224
Aqueous	Static	-	50	500	224
Aqueous	Static	-	25	500	112
*Propoxypropanol (PGPE)*					
Neat	Flow-through	200 (0–3 h); 40 (3–24 h)	100	200	177
Neat	Static	-	100	200	177
Aqueous	Static	-	75	500	331.9
Aqueous	Flow-through	200 (0–3 h); 40 (3–24 h)	50	500	221.3
Aqueous	Static	-	50	500	221.3
Aqueous	Static	-	25	500	110.6

**Table 3:** Recovery for each component of the flow-through diffusion cells expressed as the percentage of applied dose.

Conditions	Donor Chamber (%)	Receptor Chamber (%)	Receptor Chamber over 24 h * (%)	Recovery (%)
PGEE neat	0.0003	0.5	16.8	17.3
PGEE 50:50 (aq)	0.02	1.1	44.0	45.1
PGPE neat	0.18	0.3	9.4	9.9
PGPE 50:50 (aq)	0.05	0.4	24.1	24.6

* Sum of receptor fluid collected at different time points during the 24 h of exposure.

## Data Availability

The raw data supporting the conclusions of this article will be made available by the authors on request.

## Abbreviations

The following abbreviations are used in this manuscript:

PGE	Propylene glycol ether
EGE	Ethylene glycol ether
PGEE	Ethoxypropanol
PGPE	Propoxypropanol
EGBE	Ethylene glycol butyl ether
DPGME	Dipropylene glycol methyl ether
CLP	Classification, labeling and packaging of substances and mixtures
J	Permeability rate
Kp	Permeability coefficient
Tlag	Lag time
OECD	Organization for Economic Co-operation and Development
CHUV	Lausanne University Hospital
DAL	Department of Musculoskeletal Medicine
CER-VD	Commission cantonale d’éthique de la recherche sur l’être humain
TEWL	Trans epidermal water loss
GC-MS/MS	Gas-chromatography tandem mass spectrometry
